# Time trends in life expectancy of people with severe mental illness in Scotland, 2000–2019: population-based study

**DOI:** 10.1192/bjo.2025.49

**Published:** 2025-05-13

**Authors:** Kelly J. Fleetwood, Raied Alotaibi, Stine H. Scheuer, Daniel J. Smith, Sarah H. Wild, Caroline A. Jackson

**Affiliations:** Usher Institute, University of Edinburgh, Edinburgh, UK; Clinical and Translational Research, Steno Diabetes Centre Copenhagen, Herlev, Denmark; Development, Novo Nordisk A/S, Søborg, Denmark; Centre for Clinical Brain Sciences, University of Edinburgh, Edinburgh, UK

**Keywords:** Schizophrenia, bipolar disorder, depression, life expectancy

## Abstract

**Background:**

People with severe mental illness (SMI) have a higher risk of premature mortality than the general population.

**Aims:**

To investigate whether the life expectancy gap for people with SMI is widening, by determining time trends in excess life-years lost.

**Method:**

This population-based study included people with SMI (schizophrenia, bipolar disorder and major depression) alive on 1 January 2000. We ascertained SMI from psychiatric hospital admission records (1981–2019), and deaths via linkage to the national death register (2000–2019). We used the Life Years Lost (LYL) method to estimate LYL by SMI and sex, compared LYL to the Scottish population and assessed trends over 18 3-year rolling periods.

**Results:**

We included 28 797 people with schizophrenia, 16 657 with bipolar disorder and 72 504 with major depression. Between 2000 and 2019, life expectancy increased in the Scottish population but the gap widened for people with schizophrenia. For 2000–2002, men and women with schizophrenia lost an excess 9.4 (95% CI 8.5–10.3) and 8.2 (95% CI 7.4–9.0) life-years, respectively, compared with the general population. In 2017–2019, this increased to 11.8 (95% CI 10.9–12.7) and 11.1 (95% CI 10.0–12.1). The life expectancy gap was lower for bipolar disorder and depression and unchanged over time.

**Conclusions:**

The life expectancy gap in people with SMI persisted or widened from 2000 to 2019. Addressing this entrenched disparity requires equitable social, economic and health policies, healthcare re-structure and improved resourcing, and investment in interventions for primary and secondary prevention of SMI and associated comorbidities.

Improvement in life expectancy in the UK general population has stalled since 2010,^1–3^ and has been accompanied by widening gaps in life expectancy, such as that observed for people living in the most versus least deprived areas.^1,3,4^ It is, however, unclear how such trends have affected the long-recognised, yet stubbornly high, premature mortality among people with severe mental illness (SMI). Some studies, including from the UK, report a widening in the relative mortality gap among those with SMI compared with the general population in recent decades.^5–8^ However, from a public health impact perspective, it is important to examine changes in absolute measures of excess mortality, such as life expectancy. Recent meta-analyses report life expectancy disparities of 14.5 years for schizophrenia,^9^ 12.9 years for bipolar disorder^10^ and 7–19 years for clinically diagnosed depression,^11^ albeit with considerable variation across studies. Information on the life expectancy of people with SMI in the UK is limited and based largely on data from South London,^12–14^ with the only national estimates derived from a Scottish study for the period 1986 to 2010.^15^

## Time trends in life expectancy

Only one UK-based study, from South London, has examined time trends in life expectancy for people with bipolar disorder and schizophrenia, with the authors reporting an overall small narrowing of life expectancy disparities between 2008–2012 and 2013–2017, with variation by disorder and gender.^12^ Studies outside the UK are conflicting, suggesting widening,^16^ narrowing^17^ or no change^18–21^ in mortality gaps over time. Many of these studies included non-contemporaneous time periods, and most included composite mood disorder groups, with few examining bipolar disorder and major depression separately. Additionally, most existing studies have assumed a fixed age at onset of SMI (often at birth or age 15 years), which does not account for the distribution of age at onset of SMI and potentially biases life expectancy estimates.^22^

## Aims

In light of evolving methodological approaches to calculating life expectancy^22^ and the limited study of life expectancy time trends in people with SMI in the UK, we aimed to determine life-years lost among people with SMI (defined here as schizophrenia, bipolar disorder or major depression) in Scotland, stratified by sex, from 2000 to 2019 for all deaths and natural and unnatural deaths. We also assessed whether the life expectancy gap for people with SMI versus the general population has changed over time.

## Method

We have reported this study in accordance with the Reporting of Studies Conducted using Observational Routinely-Collected Health Data (RECORD) statement.^23^

We obtained approval to conduct this study with pseudonymised, non-consented data from the Public Benefit and Privacy Panel of National Health Service National Services Scotland, reference number 1516-0626.

### Data

The electronic Data Research and Innovation Service (eDRIS) provided us with records from the Scottish Morbidity Record SMR04 data-set, which includes information on admissions to all National Health Service psychiatric hospitals in Scotland.^24^ We were provided with records from adults aged at least 18 years old, who were admitted to hospital from 1 January 1981 to 31 December 2019. SMR04 includes admission date, age, sex, area-based deprivation (measured by the Carstairs Index^25^) at the time of admission and up to six discharge diagnoses. Diagnoses were coded with the ICD-9 up to March 1996, and ICD-10 from April 1996. The eDRIS team linked the SMR04 data via individuals’ unique community health index number to National Records of Scotland (NRS) death records, which include date and cause of death from the death certificate. We sourced life expectancy data for the whole Scottish population from publicly available life tables produced by NRS.^26^

### Severe mental illness

We identified people with a psychiatric hospital diagnosis of schizophrenia (ICD-10 codes F20 and F25; ICD-9 codes 295.0–295.3 and 295.6–295.9), bipolar disorder (ICD-10 codes F30–F31; ICD-9 codes 296.0 and 296.2–296.6) or major depression (ICD-10 codes F32–F33; ICD-9 codes 296.1, 298.0, 300.4 and 311) from the SMR04 records. For people with a record of more than one SMI, we used their most severe condition, with schizophrenia considered the most severe, followed by bipolar disorder and major depression. Our SMI cohort included adults aged 18 years or older who had a psychiatric hospital diagnosis of SMI and were alive on 1 January 2000. Individuals entered the cohort follow-up period either on 1 January 2000, if they had an earlier diagnosis of SMI, or on their date of first diagnosis of SMI. For people with a diagnosis of more than one SMI, we used the date of first SMI diagnosis (irrespective of condition), since more severe conditions might initially present as less severe conditions. To evaluate the impact of this approach, we also conducted a sensitivity analysis where the date of diagnosis was instead defined as the earliest record of the most severe SMI.

### Cause of death

We used ICD-10 codes to identify natural and unnatural causes of death. Unnatural causes of death included suicide, self-harm and injuries of undetermined intent (X60–X84, Y10–Y34, Y87.0 and Y87.2), accidents (V01–X59, Y85–Y86) and all other external causes (Y40–Y84, X85–Y09, Y87.1, Y88–Y89). All other deaths were classified as natural causes.

### Life expectancy

Many studies of life expectancy for people with SMI have assumed a fixed age of mental health disorder onset, e.g. 15 years, using population mortality rates up to the fixed age and condition-specific mortality rates beyond.^12–14,16,27,28^ However, a limitation of this approach is that it does not account for variation in the age at onset and can lead to biased estimates of life expectancy and years of life lost due to the condition.^22^ In this study we used a recently developed method for calculating the years of life lost due to a condition, termed the Life Years Lost (LYL) method.^22,29^ Unlike earlier methods, this accounts for variation in age at onset of the condition by estimating the LYL for each possible age at onset up to a set maximum age, and calculating a weighted average of LYL across the ages of onset. Where the probability of survival beyond the set maximum age is low, the LYL can be interpreted as the average reduction in life expectancy due to the condition. We chose a maximum age of 95 years for consistency with previous studies^19,21,30^; few people in Scotland survive beyond this age.^31^

### Statistical analysis

For each combination of SMI and sex, we summarised age, calendar year and area-based deprivation at the time of first SMI diagnosis as well as the number of deaths, age at death and cause of death.

We calculated LYL separately for each SMI, by sex. To evaluate trends over time, we calculated rolling averages of LYL for 18 overlapping 3-year periods from 2000–2002 to 2017–2019, aligning with time periods for official estimates of life expectancy in the Scottish population.^26^ For each period, we created a subcohort of people who were alive at the start of the 3-year period and had a first record of SMI diagnosis either before or during the 3-year period. We defined age at start of follow-up as age on 1 January of the index year for people with an SMI diagnosis record before the start of the 3-year period; or age at diagnosis for people with a first-recorded SMI diagnosis during the 3-year period. We identified deaths during the 3-year period, and defined age at the end of follow-up as age on 31 December of the final year for people who did not die within the 3-year period; and age at the time of death for people who died within the period. For each combination of SMI, sex and period, we calculated overall LYL from age at onset of SMI based on years lost after age 18 years and before age 95 years, and decomposed the results into LYL due to natural and unnatural causes.^32^ For each individual year of age from 18 to 94 years, we estimated the remaining life expectancy overall and by natural or unnatural cause of death, and used bootstrapping with 500 iterations to estimate confidence intervals. Finally, we estimated overall LYL by weighting the age-specific estimates of remaining life expectancy according to age at first-recorded SMI diagnosis. For each combination of SMI and sex, we calculated the age at SMI diagnosis distribution based on the entire cohort, rather than the period-specific cohort. This approach ensured that the distribution was based on a large sample, allowing trends in LYL over time to solely reflect changes in life expectancy rather than changes in both life expectancy and the distribution of age at SMI diagnosis.

We calculated excess LYL for people with SMI compared with the Scottish population, for each combination of SMI, sex, 3-year calendar period and age between 18 and 94 years, using data from the Scottish life tables. As above, the overall excess LYL was estimated by weighting the age-specific excess LYLs by the age at SMI diagnosis distribution. Hence, the overall excess LYL can be interpreted as the additional LYLs from diagnosis by people with an SMI relative to a population of the same age and sex.

To examine survival patterns by age, we plotted survival curves for each SMI and the Scottish population by sex for the earliest (2000–2002) and latest (2017–2019) periods. We plotted survival curves from age 35 years, to ensure a sufficient number of people with SMI at risk at each age.

Finally, as a sensitivity analysis, we repeated the LYL and excess LYL calculations, defining the date of diagnosis for people with more than one SMI as the earliest record of the most severe SMI (instead of the earliest record of SMI). Since depression is the least severe condition in our hierarchy, people categorised into the depression group do not have another SMI diagnosis. Hence the change in the definition of the diagnosis date does not affect the estimates of LYL and excess LYL for depression.

We used R for Windows, version 4.2.0 (R Foundation for Statistical Computing, Vienna, Austria; see https://cran.r-project.org/bin/windows/base/old/4.2.0/) to conduct all data preparation, analysis and visualisation, and specifically the R package lillies^22^ to implement the LYL method. R scripts for the analyses presented in the paper are available at: https://github.com/kjfleetwood/le_smi_scot (doi: 10.5281/zenodo.13382919).

## Results

**Fig. 1:**
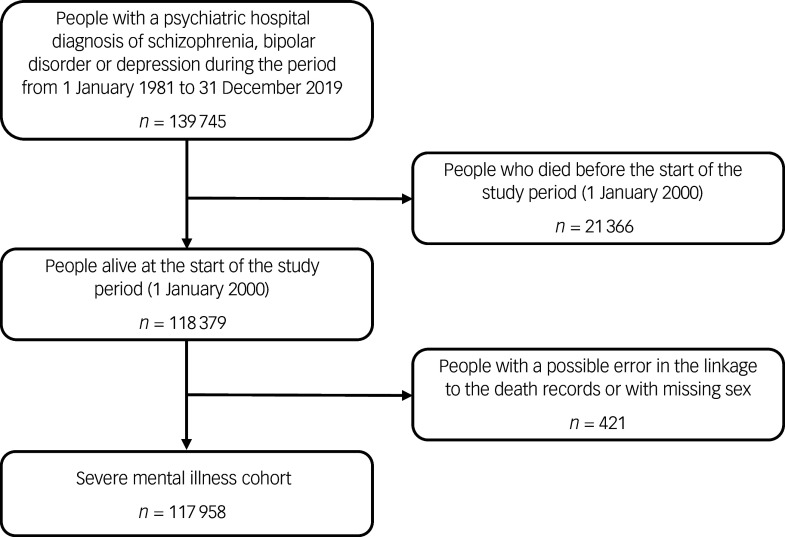
Flow diagram for the severe mental illness cohort.

We included 117 958 people with SMI (Fig. 1), of whom 28 797 (24.4%) had schizophrenia, 16 657 (14.1%) had bipolar disorder and 72 504 (61.5%) had major depression. Age at first-recorded SMI diagnosis was lowest among people with schizophrenia (median 31 years for men and 37 years for women). People with schizophrenia or major depression generally lived in more deprived areas, but there was no deprivation pattern for bipolar disorder. Approximately a third of the cohort died during the study period, with median age at death lower for people with schizophrenia (60 and 71 years for men and women, respectively) than those with bipolar disorder or major depression (Table 1).

**Table 1:** Baseline characteristics by severe mental illness and sex, and deaths during 2000–2019

Characteristics	Schizophrenia, male (*n* = 17 492)	Schizophrenia, female (*n* = 11 305)	Bipolar disorder, male (*n* = 6420)	Bipolar disorder, female (*n* = 10 237)	Major depression, male (*n* = 29 379)	Major depression, female (*n* = 43 125)
Age at first recorded SMI diagnosis, median (Q1, Q3)	31.0 (24.0, 41.0)	37.0 (28.0, 51.0)	40.0 (29.0, 53.0)	41.0 (30.0, 54.0)	42.0 (31.0, 56.0)	43.0 (31.0, 59.0)
Year of first recorded SMI diagnosis^a^						
1981–1989	4710 (26.9%)	4274 (37.8%)	1430 (22.3%)	3116 (30.4%)	4795 (16.3%)	10749 (24.9%)
1990–1999	5313 (30.4%)	3083 (27.3%)	1770 (27.6%)	2656 (25.9%)	8520 (29.0%)	13008 (30.2%)
2000–2009	4512 (25.8%)	2383 (21.1%)	1753 (27.3%)	2409 (23.5%)	9273 (31.6%)	11983 (27.8%)
2010–2019	2957 (16.9%)	1565 (13.8%)	1467 (22.9%)	2056 (20.1%)	6791 (23.1%)	7385 (17.1%)
Carstairs Index quintile at time of SMI diagnosis						
1 (most deprived)	5026 (28.7%)	3049 (27.0%)	1251 (19.5%)	2078 (20.3%)	7357 (25.0%)	10841 (25.1%)
2	3662 (20.9%)	2374 (21.0%)	1233 (19.2%)	2006 (19.6%)	6098 (20.8%)	9049 (21.0%)
3	2988 (17.1%)	2042 (18.1%)	1181 (18.4%)	1930 (18.9%)	5650 (19.2%)	8281 (19.2%)
4	2502 (14.3%)	1793 (15.9%)	1147 (17.9%)	1861 (18.2%)	5061 (17.2%)	7317 (17.0%)
5 (least deprived)	2050 (11.7%)	1512 (13.4%)	1261 (19.6%)	1959 (19.1%)	4301 (14.6%)	6649 (15.4%)
Missing	1264 (7.2%)	535 (4.7%)	347 (5.4%)	403 (3.9%)	912 (3.1%)	988 (2.3%)
Deaths, *n* (%)	5026 (28.7%)	3991 (35.3%)	1966 (30.6%)	3270 (31.9%)	10341 (35.2%)	15467 (35.9%)
Age at death, median (interquartile range)	60.0 (47.0–71.0)	71.0 (60.0–80.0)	69.0 (57.0–78.0)	75.0 (65.0–83.0)	70.0 (55.0–80.0)	77.0 (64.0–85.0)
Cause of death, *n* (% of deaths)						
Natural	4127 (82.1%)	3648 (91.4%)	1691 (86.0%)	2962 (90.6%)	8765 (84.8%)	14126 (91.3%)
Unnatural	899 (17.9%)	343 (8.6%)	275 (14.0%)	308 (9.4%)	1576 (15.2%)	1341 (8.7%)

SMI, severe mental illness.

a . Indicates year of first psychiatric hospital record of SMI.

**Fig. 2:**
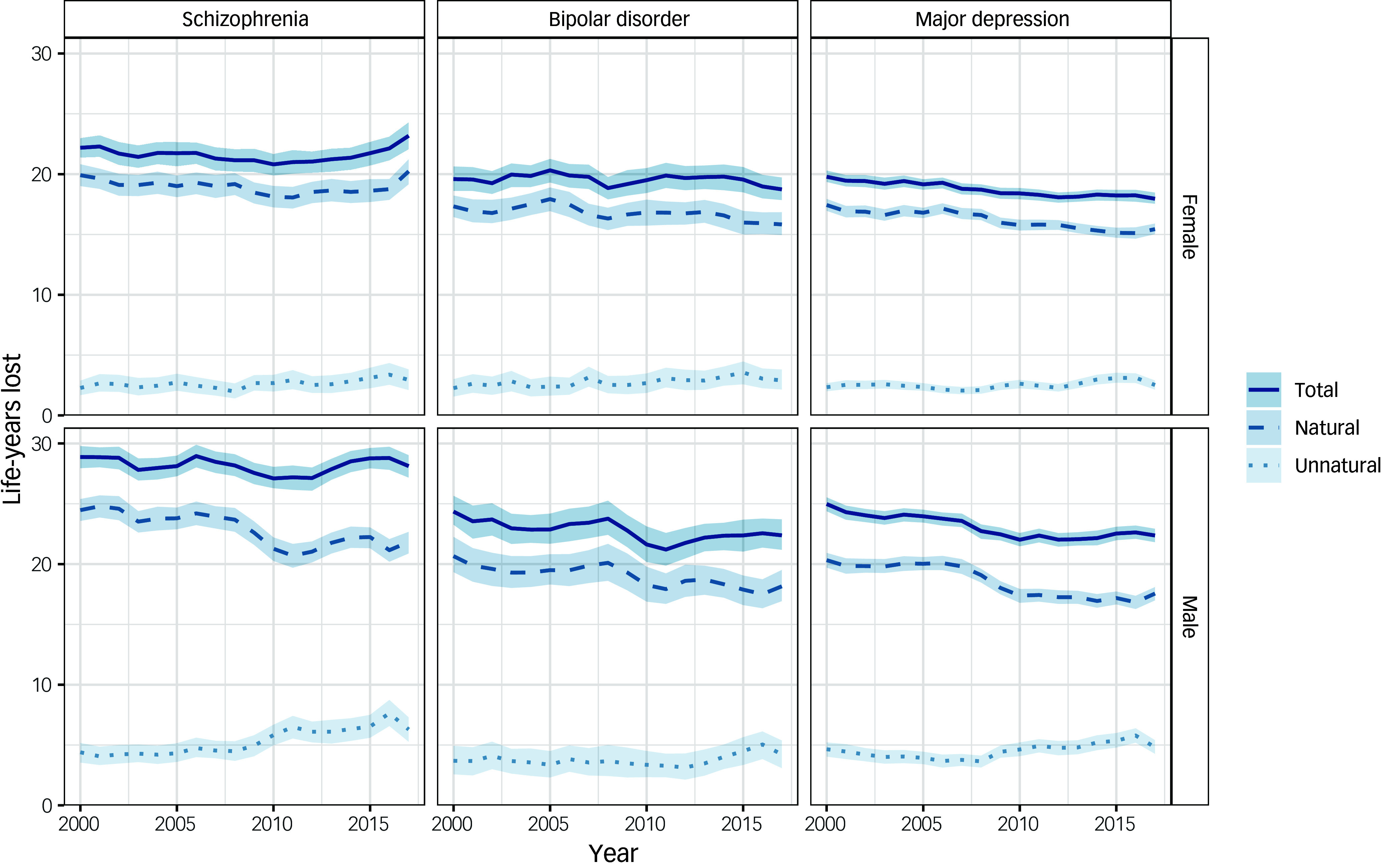
Life-years lost for all deaths and natural and unnatural deaths among people in Scotland with each severe mental illness, stratified by sex (rolling 3-year averages between 2000 and 2019). Life-years lost indicates overall life-years lost from age at onset of SMI based on years lost after age 18 years and before age 95 years. Year shown is at the start of 3-year period.

Across all SMIs, more life-years were lost due to natural rather than unnatural causes (Fig. 2, Supplementary Table 1). For schizophrenia, there was no evidence of a change in life expectancy between 2000 and 2019. Compared with a maximum age of 95 years, men with schizophrenia lost an average of 28.9 (95% CI 28.0–29.8) years in 2000–2002 and 28.1 (95% CI 27.2–29.0) years in 2017–2019; women lost an average of 22.2 (95% CI 21.4–23.0) years in 2000–2002 and 23.2 (95% CI 22.1–24.3) years in 2017–2019. For men with schizophrenia, LYL due to natural causes decreased from 24.5 (95% CI 23.6–25.4) years in 2000–2002 to 21.8 (95% CI 20.9–22.7) years in 2017–2019, whereas LYL due to unnatural causes increased from 4.4 (95% CI 3.5–5.2) years to 6.3 (95% CI 5.3–7.3) years. For women with schizophrenia, there was no evidence of a change in LYL due to either natural or unnatural causes.

Life expectancy improved among both men and women with major depression between 2000 and 2019. Compared with a maximum age of 95 years, men with major depression lost an average of 25.0 (95% CI 24.4–25.5) years in 2000–2002 and 22.4 (95% CI 21.8–22.9) years in 2017–2019, whereas women lost an average of 19.8 (95% CI 19.4–20.3) years in 2000–2002 and 18.0 (95% CI 17.5–18.5) years in 2017–2019. The pattern for people with bipolar disorder was less clear given the smaller numbers in this group. For both men and women, life expectancy may have improved slightly between 2000 and 2019, but the confidence intervals overlapped. Compared with a maximum age of 95 years, men with bipolar disorder lost an average of 24.4 (95% CI 23.3–25.7) years in 2000–2002 and 22.4 (95% CI 21.2–23.7) years in 2017–2019, whereas women lost an average of 19.6 (95% CI 18.6–20.6) years in 2000–2002 and 18.7 (95% CI 17.9–19.7) years in 2017–2019. Improvements in life expectancy in those with major depression and bipolar disorder were driven by a reduction in LYL due to natural rather than unnatural causes of death.

**Fig. 3:**
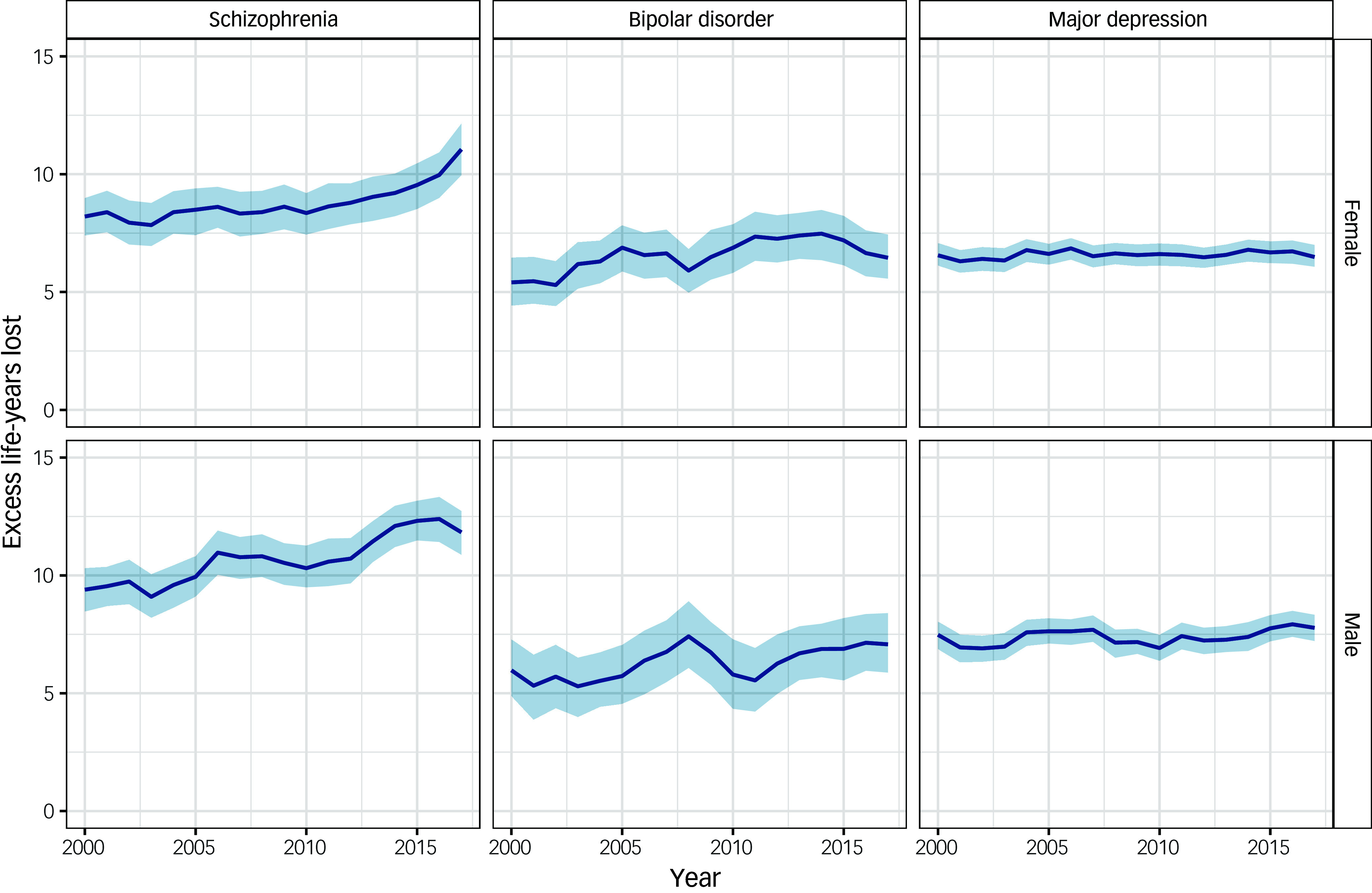
Excess life-years lost in people with a severe mental illness compared with the Scottish population, stratified by sex (rolling 3-year averages between 2000 and 2019). Year shown is at the start of 3-year period.

Overall, life expectancy in the Scottish population increased between 2000 and 2019.^33^ For men, life expectancy increased from 73.3 years in 2000–2002 to 77.1 years in 2017–2019, whereas for women it increased from 78.8 years in 2000–2002 to 81.1 years in 2017–2019. Compared with the Scottish population, people with each SMI had lower life expectancy (Fig. 3; Supplementary Table 2). The gap in life expectancy for people with schizophrenia increased between 2000 and 2019. In 2000–2002, men with schizophrenia lost 9.4 (95% CI 8.5–10.3) excess life-years from diagnosis compared with men of the same age from the Scottish population. This gap had increased by 2017–2019, with men with schizophrenia losing 11.8 (95% CI 10.9–12.7) excess life-years. Similarly, women with schizophrenia lost 8.2 (95% CI 7.4–9.0) excess life-years in 2000–2002 and 11.1 (95% CI 10.0–12.1) excess life-years in 2017–2019.

For bipolar disorder and major depression, there was no evidence of a change in the life expectancy gap relative to the Scottish population between 2000 and 2019. Men with bipolar disorder lost 6.0 (95% CI 4.9–7.3) excess life-years in 2000–2002 and 7.1 (95% CI 5.9–8.4) excess life-years in 2017–2019. Women with bipolar disorder lost 5.4 (95% CI 4.4–6.5) excess life-years in 2000–2002 and 6.5 (95% CI 5.6–7.4) excess life-years in 2017–2019. The gap was similar for people with major depression, with men losing 7.5 (95% CI 6.9–8.0) excess life-years in 2000–2002 and 7.8 (95% CI 7.2–8.3) excess life-years in 2017–2019. Women with major depression lost 6.6 (95% CI 6.1–7.1) excess life-years in 2000–2002 and 6.5 (95% CI 6.1–7.0) excess life-years in 2017–2019.

In both the earliest (2000–2002) and most recent (2017–2019) periods, differences in survival between the Scottish population and people with SMI are evident across the lifespan from 35 to 95 years (Supplementary Fig. 1).

The results of the sensitivity analysis where date of diagnosis for people with more than one SMI was defined as the earliest record of the most severe SMI are consistent with the results of the primary analysis (Supplementary Figs 2–3; Supplementary Table 3–4). Between 2000 and 2019, the life expectancy gap widened for people with schizophrenia, but there was no evidence of a change for bipolar disorder and depression.

## Discussion

### Summary of findings

Our findings reveal persistent large disparities in life expectancy of those with SMI compared with the general population across the lifespan. In Scotland, between 2000 and 2019, life expectancy in the general population increased. Although life expectancy similarly improved among those with a psychiatric hospital admission record for major depression, the life expectancy gap did not narrow. In contrast, the gap for people with schizophrenia appears to have widened. Among men with schizophrenia, LYL due to natural causes decreased, whereas LYL due to unnatural causes increased, with no changes observed among women. The pattern is less clear for bipolar disorder and, although life expectancy may have improved slightly, there is no evidence that the gap is narrowing.

### Comparison with other studies

Our estimates of the life expectancy gap for each of schizophrenia, bipolar disorder and major depression are lower than reported in some previous studies, including UK studies.^9,10,12,15^ This may reflect differences in study setting, period and methodology, including our adoption of the new LYL method, which takes account of the variation in age at onset of psychiatric illness, providing more conservative life expectancy estimates.^22^ Our estimates generally align with other recent studies from Denmark and Hong Kong that also used the LYL method.^18–21,30^

In contrast to our study, previous contemporaneous studies from Denmark and Hong Kong employing the LYL method found no change in the gap over time for people with schizophrenia compared with the general population.^19–21^ This could partly reflect differences in ascertainment method, with Danish studies defining schizophrenia based on both out-patient and in-patient hospital records. Where mood disorders were analysed, studies did not disaggregate bipolar disorder and major depression,^20^ but estimates of life expectancy and lack of change in the gap over time aligned with our findings. Interestingly, where examined in previous Danish studies, authors found that reductions in unnatural causes of death were offset by increasing deaths from natural causes.^18–20^ This contrasts with our findings, where we generally found a decrease in LYL from natural causes of death and no change or an increase in LYL from unnatural causes of deaths. This may reflect the underfunding of mental health services in the UK during this time period.^34^ The only other contemporary UK-based study found a narrowing of the life expectancy gap from 2008–2012 to 2013–2017 for all SMI disorders combined^12^; although confidence intervals of estimates for the two periods overlapped, and this narrowing was not observed across all gender and mental disorder combinations. The study also included a single highly urbanised area, limiting generalisability to the rest of England and the wider UK, and the comparator was UK-wide life expectancy rather than life expectancy of the defined geographical area of the study population. Our findings differ from those of two studies based on earlier decades of data from Australia^16^ and Nordic countries,^17^ but apparent inconsistencies may reflect the differing time periods and settings, health systems and study methodology.

Mortality rate ratios in people with schizophrenia and bipolar disorder relative to that of the general population have been reported to be widening between 2000 and 2014 in the UK.^5^ Relative measures are sensitive to decreases in the reference population mortality rates over time, resulting in larger relative differences between groups, but are essentially consistent with the changes in absolute differences in life expectancy over time that we have reported.

### Strengths and limitations

A key strength of our study is the use of recent national level data spanning two decades, which enabled assessment of change in life expectancy over a substantial period of time with stratification by sex. Given the use of whole population data during a long study period, we were able to analyse life expectancy by individual SMI, and to distinguish between bipolar disorder and major depression, which have often previously been analysed as a composite group of all mood disorders, potentially masking differences between disorders. Finally, we adopted the recently introduced LYL method that accounts for the age of diagnosis distribution and allows decomposition of LYL by cause of death. Given there is sometimes a delay between onset of psychiatric disorder and diagnosis, the true gap in life expectancy for those with SMI is likely to lie between estimates generated by this more conservative approach and earlier, less conservative, methods.

Our study has some limitations. Ascertainment of SMI through hospital admission data means we are likely to have included people with SMI at the more severe end of the spectrum or with particularly challenging/complex health and social care needs, and our findings may not be generalisable to all people with SMI. Our study included people with both prevalent SMI (diagnosed before 2000) and incident SMI (diagnosed from 2000). Previous studies have indicated that individuals with SMI have higher mortality following discharge from psychiatric hospital,^35^ and hence our inclusion of prevalent cases may underestimate mortality in people with SMI. Similarly, by using first hospital admission date as a proxy of diagnosis date, our estimates of life expectancy from disease onset may be slightly underestimated. Our findings on the widening life expectancy gap for schizophrenia may reflect changes in practice related to psychiatric hospital admission in Scotland, with number of psychiatric hospital admissions having steadily decreased from around 30 000 in 2000 to around 20 000 from 2010 onward.^36^ It is possible that people admitted in later years have more severe illness than those admitted in earlier years, which may contribute to the widening gap. Although we included nationwide data, the number of people included with bipolar disorder was smaller than in the other groups, and LYL and excess LYL estimates were therefore more uncertain. Also, we did not have information on emigration, and so people whose death was registered outside Scotland would have been lost to follow-up. Although emigration from Scotland is relatively low^37^ (and likely to be lower among those with SMI, because of a healthy immigrant effect), we may have underascertained deaths in those with SMI, thus underestimating differences in life expectancy. Unfortunately, we did not have access to data for the period 2020–2024, and so cannot comment on mental health disparities in life expectancy during these most recent years, which may have worsened as a consequence of the COVID-19 pandemic, given reports of higher COVID-19 mortality in people with SMI.^38^ Finally, a number of questions were beyond the scope of our study and warrant investigation. These include further examination of the role of deprivation, with previous studies indicating an effect of SMI on excess mortality that is over and above that of deprivation^14^; closer investigation of changes in cause-specific mortality over time, particularly alcohol-related deaths^11^; and the role of changes in mental health services (with fewer in-patient beds available).

### Implications

Excess deaths due to natural causes account for much of the observed premature mortality in people with SMI in both our study and a Danish study,^18^ reflecting the poorer physical health of people with SMI, in whom there is a high burden of multimorbidity.^39^ The cause of this premature mortality is complex, multifactorial and multi-level,^40,41^ with key risk factors operating at the individual through to societal level, and encompassing modifiable lifestyle behaviours, such as smoking, overweight/obesity and physical inactivity; multimorbidity and substance misuse; psychological factors; socioeconomic disadvantage; healthcare access, including year-on-year underfunding of mental health services and diagnostic overshadowing; iatrogenic harms of some psychotropic medication; structural inequalities; and stigma and marginalisation.^41^ Addressing these complex causes requires multi-level and synergistic solutions.^40,41^ These have recently been structured around three key principles: integration of mental and physical healthcare, prioritisation of prevention of physical disease while strengthening treatment of mental illness, and optimisation of intervention synergies across social-ecological levels.^41^ Current health system structures generally create disconnected care that particularly disadvantages patients with complex needs, such as those with SMI. In particular, there is a need for better integration across primary, secondary and community healthcare settings and between generalist and specialist providers, along with coordinated support from social care and allied health professionals.^40,42^ In addition to regular physical health monitoring in those with SMI, a greater focus on primary prevention strategies is required to tackle key modifiable risk behaviours in this group.^43^

In conclusion, our national study highlights the persistent and potentially widening gap in life expectancy experienced by people with SMI. This entrenched disparity reflects ingrained inequalities at multiple levels that affect the health and lifespan of this marginalised subgroup of the population. Future success in narrowing this shameful gap relies on government commitment to introducing equitable social, economic and health policies, significant healthcare re-structure and improved resourcing, and investment in tailored, well-implemented and appropriately evaluated interventions and strategies for primary and secondary prevention of SMI and associated comorbidities.

## Supporting information

Fleetwood et al. supplementary material 1 Fleetwood et al. supplementary material

Fleetwood et al. supplementary material 2 Fleetwood et al. supplementary material

## Data Availability

Researchers can request access to the Scottish records used in this analysis by contacting the electronic Data Research and Innovation Service (eDRIS). Details of the available data-sets and the application process are available from https://publichealthscotland.scot/services/data-research-and-innovation-services/electronic-data-research-and-innovation-service-edris.

## Supplementary material

The supplementary material can be found at https://doi.org/10.1192/bjo.2025.49
