# Supplementary material for: Time trends in life expectancy of people with severe mental illness in Scotland, 2000–2019: population-based study
Source: BJPsych Open. 2025 May 13;11(3):e103. doi: 10.1192/bjo.2025.49 (PMC12089813; doi:10.1192/bjo.2025.49)
Supplement: Fleetwood et al. supplementary material 1 — Fleetwood et al. supplementary material [file S2056472425000493sup001.docx]

Time-trends in life expectancy of people with severe mental illness in Scotland, 2000-2019: a population-based study

Kelly J Fleetwood^1^, Raied Alotaibi^1^, Stine H Scheuer^2,3^, Daniel J Smith^4^, Sarah H Wild^1^, Caroline A Jackson^1^

1. Usher Institute, University of Edinburgh, Edinburgh, UK
2. Steno Diabetes Centre Copenhagen, Herlev, Denmark
3. Novo Nordisk, Copenhagen, Denmark
4. Centre for Clinical Brain Sciences, University of Edinburgh, Edinburgh, UK

Correspondence to:

Kelly Fleetwood

Email: [kelly.fleetwood@ed.ac.uk](mailto:kelly.fleetwood@ed.ac.uk)

Address: Usher Institute, Usher Building, The University of Edinburgh, 5‒7 Little France Road, Edinburgh BioQuarter ‒ Gate 3, Edinburgh, EH16 4UX, UK

Table of Contents

[Supplementary Table 1: Life years lost for all deaths and natural and unnatural deaths among people in Scotland with each severe mental illness, stratified by sex (rolling three-year averages between 2000 and 2019) 3](#_Toc190357934)

[Supplementary Table 2: Excess life years lost in people with a severe mental illness in comparison to the Scottish population, stratified by sex (rolling three-year averages between 2000 and 2019) 6](#_Toc190357935)

[Supplementary Figure 1: Survival curves for the Scottish population and those with each of schizophrenia, bipolar disorder and major depression, stratified by sex, in 2000-2002 and 2017-2019 7](#_Toc190357936)

[Supplementary Figure 2: Life years lost for all deaths and natural and unnatural deaths among people in Scotland with each severe mental illness, stratified by sex (rolling three-year averages between 2000 and 2019) (sensitivity analysis with the SMI diagnosis date based on the earliest record of the most severe SMI) 8](#_Toc190357937)

[Supplementary Figure 3: Survival curves for the Scottish population and those with each of schizophrenia, bipolar disorder and major depression, stratified by sex, in 2000-2002 and 2017-2019 (sensitivity analysis with the SMI diagnosis date based on the earliest record of the most severe SMI) 9](#_Toc190357938)

[Supplementary Table 3: Life years lost for all deaths and natural and unnatural deaths among people in Scotland with each severe mental illness, stratified by sex (rolling three-year averages between 2000 and 2019) (sensitivity analysis with the SMI diagnosis date based on the earliest record of the most severe SMI) 10](#_Toc190357939)

[Supplementary Table 4: Excess life years lost in people with a severe mental illness in comparison to the Scottish population, stratified by sex (rolling three-year averages between 2000 and 2019) (sensitivity analysis with the SMI diagnosis date based on the earliest record of the most severe SMI) 13](#_Toc190357940)

Supplementary Table 1: Life years lost for all deaths and natural and unnatural deaths among people in Scotland with each severe mental illness, stratified by sex (rolling three-year averages between 2000 and 2019) 3

Supplementary Table 2: Excess life years lost in people with a severe mental illness in comparison to the Scottish population, stratified by sex (rolling three-year averages between 2000 and 2019) 6

Supplementary Figure 1: Survival curves for the Scottish population and those with each of schizophrenia, bipolar disorder and major depression, stratified by sex, in 2000-2002 and 2017-2019 7

# Supplementary Table 1: Life years lost for all deaths and natural and unnatural deaths among people in Scotland with each severe mental illness, stratified by sex (rolling three-year averages between 2000 and 2019)

|  |  |  | **SMI** | | |
| --- | --- | --- | --- | --- | --- |
| **Cause of death** | **Sex** | **Period** | **Schizophrenia** | **Bipolar disorder** | **Major depression** |
| All | Male | 2000-02 | 28.9 (28.0, 29.8) | 24.4 (23.3, 25.7) | 25.0 (24.4, 25.5) |
|  |  | 2001-03 | 28.9 (28.0, 29.7) | 23.5 (22.1, 24.9) | 24.3 (23.7, 24.8) |
|  |  | 2002-04 | 28.8 (27.9, 29.7) | 23.7 (22.4, 25.0) | 24.0 (23.5, 24.6) |
|  |  | 2003-05 | 27.8 (26.9, 28.8) | 23.0 (21.7, 24.2) | 23.8 (23.3, 24.4) |
|  |  | 2004-06 | 28.0 (27.0, 28.8) | 22.9 (21.8, 24.1) | 24.1 (23.5, 24.6) |
|  |  | 2005-07 | 28.1 (27.3, 29.0) | 22.9 (21.7, 24.2) | 24.0 (23.4, 24.5) |
|  |  | 2006-08 | 29.0 (28.0, 29.9) | 23.3 (21.9, 24.6) | 23.8 (23.2, 24.3) |
|  |  | 2007-09 | 28.5 (27.6, 29.3) | 23.4 (22.1, 24.8) | 23.6 (23.1, 24.2) |
|  |  | 2008-10 | 28.2 (27.3, 29.1) | 23.8 (22.4, 25.3) | 22.7 (22.1, 23.3) |
|  |  | 2009-11 | 27.6 (26.6, 28.4) | 22.8 (21.4, 24.1) | 22.5 (22.0, 23.0) |
|  |  | 2010-12 | 27.1 (26.3, 28.1) | 21.6 (20.2, 23.1) | 22.0 (21.5, 22.6) |
|  |  | 2011-13 | 27.2 (26.2, 28.2) | 21.2 (19.9, 22.6) | 22.4 (21.8, 22.9) |
|  |  | 2012-14 | 27.1 (26.1, 28.0) | 21.8 (20.5, 23.0) | 22.0 (21.4, 22.6) |
|  |  | 2013-15 | 27.9 (27.0, 28.7) | 22.2 (21.1, 23.3) | 22.1 (21.5, 22.6) |
|  |  | 2014-16 | 28.5 (27.6, 29.4) | 22.4 (21.2, 23.4) | 22.2 (21.6, 22.8) |
|  |  | 2015-17 | 28.8 (27.9, 29.6) | 22.4 (21.0, 23.7) | 22.5 (22.0, 23.1) |
|  |  | 2016-18 | 28.8 (27.8, 29.7) | 22.6 (21.4, 23.8) | 22.6 (22.1, 23.2) |
|  |  | 2017-19 | 28.1 (27.2, 29.0) | 22.4 (21.2, 23.7) | 22.4 (21.8, 22.9) |
|  | Female | 2000-02 | 22.2 (21.4, 23.0) | 19.6 (18.6, 20.6) | 19.8 (19.4, 20.3) |
|  |  | 2001-03 | 22.3 (21.4, 23.2) | 19.6 (18.6, 20.6) | 19.5 (19.0, 19.9) |
|  |  | 2002-04 | 21.7 (20.8, 22.6) | 19.2 (18.4, 20.3) | 19.4 (18.9, 19.9) |
|  |  | 2003-05 | 21.4 (20.5, 22.4) | 20.0 (18.9, 20.9) | 19.2 (18.7, 19.7) |
|  |  | 2004-06 | 21.8 (20.8, 22.6) | 19.8 (18.9, 20.7) | 19.4 (18.9, 19.9) |
|  |  | 2005-07 | 21.7 (20.7, 22.6) | 20.3 (19.3, 21.3) | 19.2 (18.7, 19.6) |
|  |  | 2006-08 | 21.8 (20.9, 22.6) | 19.9 (18.9, 20.8) | 19.3 (18.8, 19.7) |
|  |  | 2007-09 | 21.3 (20.3, 22.2) | 19.8 (18.8, 20.8) | 18.8 (18.3, 19.2) |
|  |  | 2008-10 | 21.2 (20.2, 22.1) | 18.8 (17.9, 19.8) | 18.7 (18.3, 19.2) |
|  |  | 2009-11 | 21.2 (20.2, 22.1) | 19.2 (18.2, 20.3) | 18.4 (18.0, 18.9) |
|  |  | 2010-12 | 20.8 (19.9, 21.7) | 19.5 (18.4, 20.5) | 18.4 (17.9, 18.9) |
|  |  | 2011-13 | 21.0 (20.0, 22.0) | 19.9 (18.9, 20.9) | 18.3 (17.8, 18.7) |
|  |  | 2012-14 | 21.0 (20.1, 21.9) | 19.7 (18.7, 20.7) | 18.1 (17.6, 18.5) |
|  |  | 2013-15 | 21.2 (20.2, 22.1) | 19.8 (18.8, 20.7) | 18.1 (17.7, 18.6) |
|  |  | 2014-16 | 21.4 (20.4, 22.2) | 19.8 (18.7, 20.8) | 18.3 (17.8, 18.7) |
|  |  | 2015-17 | 21.7 (20.7, 22.7) | 19.6 (18.5, 20.6) | 18.2 (17.8, 18.7) |
|  |  | 2016-18 | 22.1 (21.2, 23.1) | 19.0 (18.0, 19.9) | 18.2 (17.7, 18.7) |
|  |  | 2017-19 | 23.2 (22.1, 24.3) | 18.7 (17.9, 19.7) | 18.0 (17.5, 18.5) |
| Natural | Male | 2000-02 | 24.5 (23.6, 25.4) | 20.7 (19.3, 22.3) | 20.3 (19.7, 20.9) |
|  |  | 2001-03 | 24.8 (23.9, 25.7) | 19.9 (18.5, 21.3) | 19.8 (19.2, 20.5) |
|  |  | 2002-04 | 24.6 (23.6, 25.6) | 19.6 (18.2, 20.9) | 19.8 (19.3, 20.4) |
|  |  | 2003-05 | 23.5 (22.6, 24.4) | 19.3 (18.0, 20.7) | 19.8 (19.3, 20.4) |
|  |  | 2004-06 | 23.8 (22.8, 24.8) | 19.3 (18.1, 20.7) | 20.1 (19.4, 20.7) |
|  |  | 2005-07 | 23.8 (22.9, 24.7) | 19.5 (18.3, 20.8) | 20.0 (19.5, 20.6) |
|  |  | 2006-08 | 24.2 (23.2, 25.2) | 19.5 (18.2, 20.8) | 20.1 (19.5, 20.7) |
|  |  | 2007-09 | 23.9 (23.0, 24.8) | 19.9 (18.4, 21.2) | 19.8 (19.2, 20.4) |
|  |  | 2008-10 | 23.7 (22.7, 24.7) | 20.1 (18.6, 21.7) | 19.1 (18.5, 19.6) |
|  |  | 2009-11 | 22.6 (21.6, 23.5) | 19.3 (17.8, 20.9) | 18.0 (17.5, 18.6) |
|  |  | 2010-12 | 21.3 (20.3, 22.2) | 18.3 (16.9, 19.8) | 17.4 (16.8, 17.9) |
|  |  | 2011-13 | 20.7 (19.7, 21.7) | 17.9 (16.7, 19.2) | 17.4 (16.9, 18.0) |
|  |  | 2012-14 | 21.0 (20.1, 21.9) | 18.6 (17.3, 19.9) | 17.3 (16.7, 17.8) |
|  |  | 2013-15 | 21.8 (20.9, 22.6) | 18.7 (17.5, 19.9) | 17.3 (16.7, 17.8) |
|  |  | 2014-16 | 22.2 (21.3, 23.1) | 18.3 (17.2, 19.6) | 16.9 (16.4, 17.5) |
|  |  | 2015-17 | 22.2 (21.3, 23.0) | 17.9 (16.6, 19.1) | 17.2 (16.7, 17.7) |
|  |  | 2016-18 | 21.2 (20.2, 22.1) | 17.5 (16.3, 18.7) | 16.8 (16.3, 17.4) |
|  |  | 2017-19 | 21.8 (20.9, 22.7) | 18.2 (16.9, 19.5) | 17.6 (17.0, 18.1) |
|  | Female | 2000-02 | 19.9 (19.0, 20.8) | 17.3 (16.4, 18.2) | 17.4 (17.0, 17.9) |
|  |  | 2001-03 | 19.6 (18.8, 20.5) | 16.9 (16.0, 17.9) | 16.9 (16.4, 17.4) |
|  |  | 2002-04 | 19.1 (18.2, 20.0) | 16.8 (15.9, 17.9) | 16.9 (16.4, 17.4) |
|  |  | 2003-05 | 19.1 (18.2, 19.9) | 17.1 (16.2, 18.1) | 16.6 (16.1, 17.1) |
|  |  | 2004-06 | 19.3 (18.4, 20.2) | 17.5 (16.6, 18.4) | 17.0 (16.5, 17.4) |
|  |  | 2005-07 | 19.0 (18.1, 19.9) | 17.9 (16.9, 18.9) | 16.8 (16.4, 17.2) |
|  |  | 2006-08 | 19.3 (18.3, 20.1) | 17.5 (16.4, 18.5) | 17.1 (16.7, 17.6) |
|  |  | 2007-09 | 19.0 (18.0, 19.9) | 16.6 (15.7, 17.5) | 16.7 (16.3, 17.2) |
|  |  | 2008-10 | 19.2 (18.3, 20.1) | 16.3 (15.4, 17.2) | 16.6 (16.2, 17.1) |
|  |  | 2009-11 | 18.5 (17.6, 19.3) | 16.7 (15.7, 17.6) | 16.0 (15.5, 16.4) |
|  |  | 2010-12 | 18.1 (17.2, 19.0) | 16.8 (15.7, 17.9) | 15.8 (15.3, 16.2) |
|  |  | 2011-13 | 18.1 (17.1, 18.9) | 16.8 (15.8, 17.7) | 15.8 (15.4, 16.2) |
|  |  | 2012-14 | 18.5 (17.6, 19.4) | 16.8 (15.8, 17.7) | 15.8 (15.4, 16.2) |
|  |  | 2013-15 | 18.6 (17.7, 19.6) | 16.9 (16.0, 17.9) | 15.5 (15.1, 15.9) |
|  |  | 2014-16 | 18.5 (17.6, 19.4) | 16.6 (15.5, 17.5) | 15.3 (14.9, 15.7) |
|  |  | 2015-17 | 18.6 (17.7, 19.6) | 16.0 (15.0, 17.0) | 15.1 (14.7, 15.6) |
|  |  | 2016-18 | 18.8 (17.9, 19.6) | 15.9 (15.0, 16.8) | 15.1 (14.7, 15.6) |
|  |  | 2017-19 | 20.2 (19.2, 21.2) | 15.8 (15.0, 16.8) | 15.4 (15.0, 15.9) |
| Unnatural | Male | 2000-02 | 4.4 (3.5, 5.2) | 3.7 (2.6, 4.9) | 4.7 (4.0, 5.2) |
|  |  | 2001-03 | 4.1 (3.3, 4.9) | 3.7 (2.5, 4.8) | 4.5 (3.8, 5.1) |
|  |  | 2002-04 | 4.2 (3.5, 5.0) | 4.1 (3.0, 5.3) | 4.2 (3.7, 4.7) |
|  |  | 2003-05 | 4.3 (3.6, 5.0) | 3.7 (2.6, 4.7) | 4.0 (3.5, 4.6) |
|  |  | 2004-06 | 4.2 (3.4, 4.9) | 3.6 (2.5, 4.7) | 4.0 (3.5, 4.6) |
|  |  | 2005-07 | 4.3 (3.6, 5.2) | 3.4 (2.3, 4.5) | 3.9 (3.4, 4.4) |
|  |  | 2006-08 | 4.8 (4.0, 5.6) | 3.8 (2.8, 5.0) | 3.7 (3.1, 4.2) |
|  |  | 2007-09 | 4.5 (3.9, 5.4) | 3.6 (2.5, 4.7) | 3.8 (3.3, 4.3) |
|  |  | 2008-10 | 4.5 (3.7, 5.3) | 3.7 (2.4, 5.0) | 3.6 (3.1, 4.1) |
|  |  | 2009-11 | 5.0 (4.1, 5.8) | 3.5 (2.3, 4.8) | 4.4 (4.0, 5.0) |
|  |  | 2010-12 | 5.8 (5.0, 6.7) | 3.4 (2.3, 4.5) | 4.6 (4.1, 5.2) |
|  |  | 2011-13 | 6.5 (5.5, 7.4) | 3.3 (2.3, 4.3) | 4.9 (4.4, 5.5) |
|  |  | 2012-14 | 6.1 (5.2, 6.9) | 3.1 (2.1, 4.3) | 4.8 (4.2, 5.3) |
|  |  | 2013-15 | 6.1 (5.1, 7.1) | 3.5 (2.5, 4.6) | 4.8 (4.2, 5.4) |
|  |  | 2014-16 | 6.3 (5.3, 7.2) | 4.0 (3.0, 5.0) | 5.2 (4.6, 5.8) |
|  |  | 2015-17 | 6.5 (5.6, 7.5) | 4.5 (3.3, 5.7) | 5.3 (4.8, 6.0) |
|  |  | 2016-18 | 7.6 (6.6, 8.7) | 5.0 (3.8, 6.1) | 5.8 (5.2, 6.4) |
|  |  | 2017-19 | 6.3 (5.3, 7.3) | 4.2 (3.1, 5.4) | 4.8 (4.3, 5.4) |
|  | Female | 2000-02 | 2.3 (1.7, 2.9) | 2.3 (1.6, 3.0) | 2.3 (2.0, 2.7) |
|  |  | 2001-03 | 2.7 (2.0, 3.4) | 2.6 (1.9, 3.4) | 2.5 (2.2, 2.9) |
|  |  | 2002-04 | 2.6 (2.0, 3.4) | 2.5 (1.7, 3.2) | 2.5 (2.2, 2.9) |
|  |  | 2003-05 | 2.3 (1.7, 3.1) | 2.8 (2.0, 3.7) | 2.6 (2.2, 2.9) |
|  |  | 2004-06 | 2.5 (1.8, 3.2) | 2.3 (1.6, 3.0) | 2.5 (2.1, 2.8) |
|  |  | 2005-07 | 2.7 (2.1, 3.5) | 2.4 (1.6, 3.2) | 2.4 (2.1, 2.7) |
|  |  | 2006-08 | 2.5 (1.8, 3.2) | 2.4 (1.7, 3.1) | 2.1 (1.8, 2.5) |
|  |  | 2007-09 | 2.3 (1.6, 2.9) | 3.2 (2.4, 4.0) | 2.1 (1.8, 2.4) |
|  |  | 2008-10 | 2.0 (1.4, 2.7) | 2.5 (1.8, 3.3) | 2.1 (1.8, 2.4) |
|  |  | 2009-11 | 2.7 (2.1, 3.3) | 2.5 (1.8, 3.3) | 2.4 (2.1, 2.8) |
|  |  | 2010-12 | 2.7 (2.0, 3.4) | 2.7 (1.9, 3.5) | 2.6 (2.2, 2.9) |
|  |  | 2011-13 | 2.9 (2.2, 3.8) | 3.1 (2.3, 4.0) | 2.5 (2.1, 2.8) |
|  |  | 2012-14 | 2.5 (1.9, 3.2) | 2.9 (2.1, 3.8) | 2.3 (2.0, 2.6) |
|  |  | 2013-15 | 2.6 (1.9, 3.3) | 2.9 (2.1, 3.7) | 2.6 (2.3, 3.0) |
|  |  | 2014-16 | 2.8 (2.0, 3.5) | 3.2 (2.5, 4.0) | 3.0 (2.6, 3.4) |
|  |  | 2015-17 | 3.1 (2.2, 4.0) | 3.6 (2.6, 4.5) | 3.1 (2.7, 3.5) |
|  |  | 2016-18 | 3.4 (2.5, 4.3) | 3.0 (2.3, 3.9) | 3.1 (2.7, 3.5) |
|  |  | 2017-19 | 2.9 (2.1, 3.8) | 2.9 (2.1, 3.8) | 2.5 (2.1, 2.9) |

# Supplementary Table 2: Excess life years lost in people with a severe mental illness in comparison to the Scottish population, stratified by sex (rolling three-year averages between 2000 and 2019)

|  |  | **SMI** | | |
| --- | --- | --- | --- | --- |
| **Sex** | **Period** | **Schizophrenia** | **Bipolar disorder** | **Depression** |
| Male | 2000-02 | 9.4 (8.5, 10.3) | 6.0 (4.9, 7.3) | 7.5 (6.9, 8.0) |
|  | 2001-03 | 9.5 (8.7, 10.4) | 5.3 (3.9, 6.6) | 6.9 (6.3, 7.5) |
|  | 2002-04 | 9.7 (8.8, 10.7) | 5.7 (4.4, 7.0) | 6.9 (6.3, 7.4) |
|  | 2003-05 | 9.1 (8.2, 10.0) | 5.3 (4.0, 6.5) | 7.0 (6.4, 7.5) |
|  | 2004-06 | 9.6 (8.6, 10.4) | 5.5 (4.4, 6.7) | 7.6 (7.0, 8.1) |
|  | 2005-07 | 9.9 (9.1, 10.8) | 5.7 (4.6, 7.0) | 7.6 (7.1, 8.2) |
|  | 2006-08 | 11.0 (10.0, 11.9) | 6.4 (5.0, 7.7) | 7.6 (7.1, 8.1) |
|  | 2007-09 | 10.8 (9.9, 11.6) | 6.8 (5.5, 8.1) | 7.7 (7.2, 8.3) |
|  | 2008-10 | 10.8 (9.9, 11.7) | 7.4 (6.1, 8.9) | 7.1 (6.5, 7.7) |
|  | 2009-11 | 10.5 (9.6, 11.4) | 6.7 (5.4, 8.0) | 7.2 (6.7, 7.7) |
|  | 2010-12 | 10.3 (9.5, 11.3) | 5.8 (4.3, 7.3) | 6.9 (6.4, 7.5) |
|  | 2011-13 | 10.6 (9.5, 11.6) | 5.5 (4.2, 6.9) | 7.4 (6.9, 8.0) |
|  | 2012-14 | 10.7 (9.7, 11.6) | 6.3 (5.0, 7.5) | 7.2 (6.7, 7.8) |
|  | 2013-15 | 11.4 (10.6, 12.3) | 6.7 (5.6, 7.8) | 7.3 (6.7, 7.8) |
|  | 2014-16 | 12.1 (11.2, 13.0) | 6.9 (5.7, 7.9) | 7.4 (6.8, 8.0) |
|  | 2015-17 | 12.3 (11.5, 13.2) | 6.9 (5.5, 8.2) | 7.8 (7.2, 8.3) |
|  | 2016-18 | 12.4 (11.4, 13.3) | 7.1 (6.0, 8.4) | 7.9 (7.4, 8.5) |
|  | 2017-19 | 11.8 (10.9, 12.7) | 7.1 (5.9, 8.4) | 7.8 (7.2, 8.3) |
| Female | 2000-02 | 8.2 (7.4, 9.0) | 5.4 (4.4, 6.5) | 6.6 (6.1, 7.1) |
|  | 2001-03 | 8.4 (7.5, 9.3) | 5.5 (4.5, 6.5) | 6.3 (5.8, 6.8) |
|  | 2002-04 | 7.9 (7.0, 8.9) | 5.3 (4.4, 6.3) | 6.4 (5.9, 6.9) |
|  | 2003-05 | 7.8 (7.0, 8.8) | 6.2 (5.2, 7.1) | 6.3 (5.9, 6.9) |
|  | 2004-06 | 8.4 (7.5, 9.3) | 6.3 (5.4, 7.2) | 6.8 (6.3, 7.2) |
|  | 2005-07 | 8.5 (7.4, 9.4) | 6.9 (5.9, 7.8) | 6.6 (6.2, 7.0) |
|  | 2006-08 | 8.6 (7.7, 9.5) | 6.6 (5.6, 7.5) | 6.9 (6.4, 7.3) |
|  | 2007-09 | 8.3 (7.4, 9.3) | 6.6 (5.6, 7.6) | 6.5 (6.1, 7.0) |
|  | 2008-10 | 8.4 (7.5, 9.3) | 5.9 (5.0, 6.8) | 6.6 (6.2, 7.1) |
|  | 2009-11 | 8.6 (7.7, 9.6) | 6.5 (5.5, 7.6) | 6.6 (6.1, 7.0) |
|  | 2010-12 | 8.4 (7.4, 9.2) | 6.9 (5.8, 7.9) | 6.6 (6.1, 7.1) |
|  | 2011-13 | 8.6 (7.7, 9.6) | 7.4 (6.3, 8.4) | 6.6 (6.1, 7.0) |
|  | 2012-14 | 8.8 (7.9, 9.6) | 7.3 (6.3, 8.3) | 6.5 (6.0, 6.9) |
|  | 2013-15 | 9.0 (8.0, 9.9) | 7.4 (6.4, 8.4) | 6.6 (6.2, 7.0) |
|  | 2014-16 | 9.2 (8.2, 10.0) | 7.5 (6.4, 8.5) | 6.8 (6.3, 7.2) |
|  | 2015-17 | 9.5 (8.5, 10.5) | 7.2 (6.1, 8.2) | 6.7 (6.2, 7.1) |
|  | 2016-18 | 10.0 (9.0, 10.9) | 6.7 (5.7, 7.6) | 6.7 (6.2, 7.2) |
|  | 2017-19 | 11.1 (10.0, 12.1) | 6.5 (5.6, 7.4) | 6.5 (6.1, 7.0) |

# Supplementary Figure 1: Survival curves for the Scottish population and those with each of schizophrenia, bipolar disorder and major depression, stratified by sex, in 2000-2002 and 2017-2019


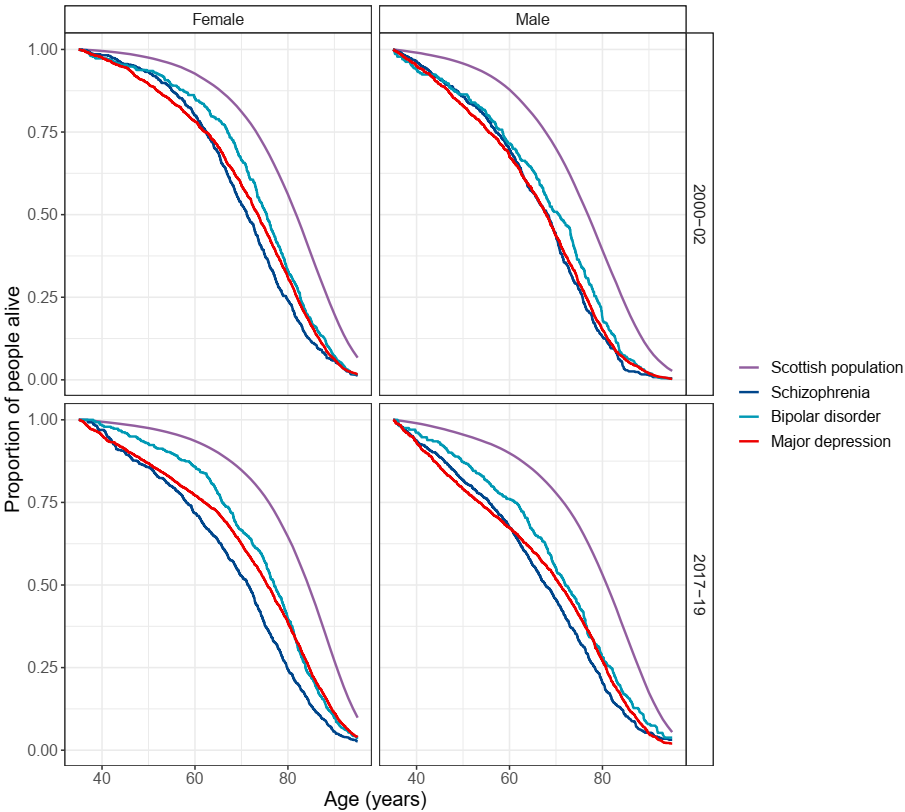


# Supplementary Figure 2: Life years lost for all deaths and natural and unnatural deaths among people in Scotland with each severe mental illness, stratified by sex (rolling three-year averages between 2000 and 2019) (sensitivity analysis with the SMI diagnosis date based on the earliest record of the most severe SMI)


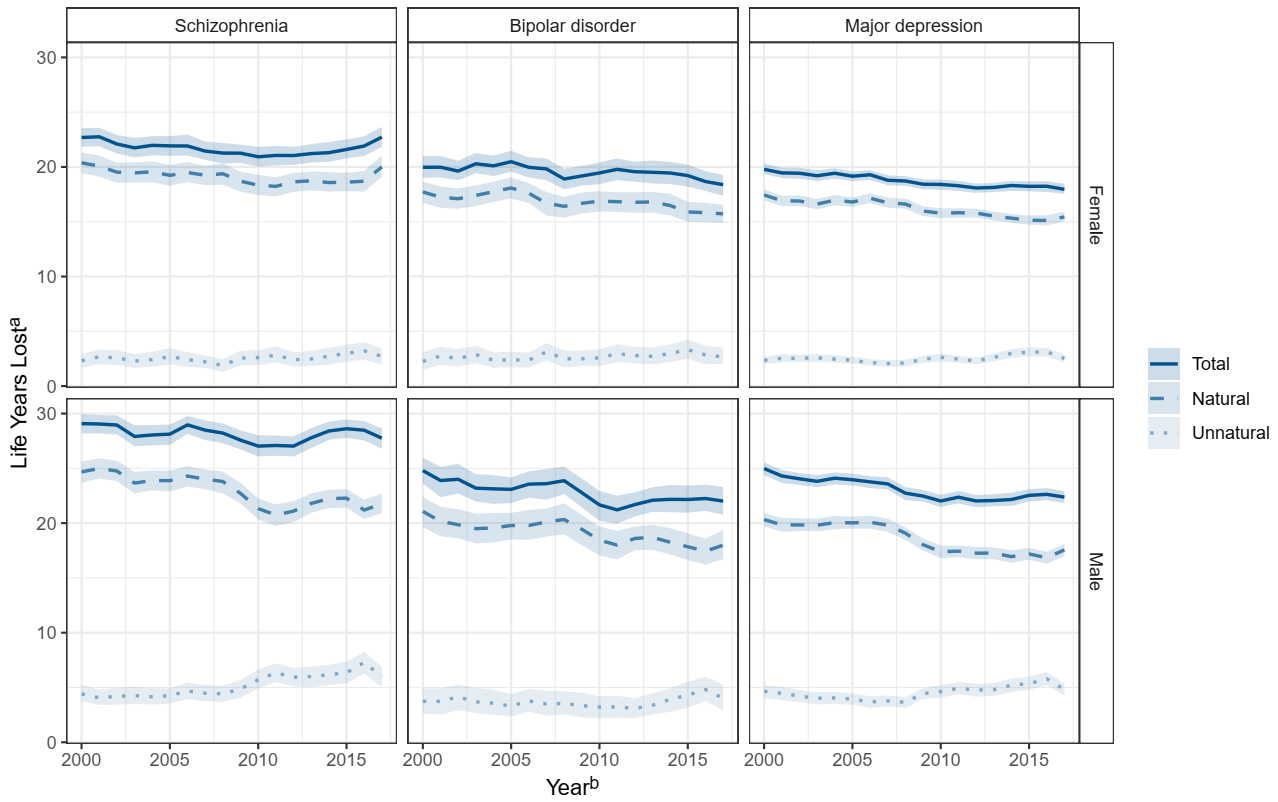


a. overall life years lost from age of onset of SMI based on years lost after age 18 years and before age 95 years

b. at the start of three-year period

# Supplementary Figure 3: Survival curves for the Scottish population and those with each of schizophrenia, bipolar disorder and major depression, stratified by sex, in 2000-2002 and 2017-2019 (sensitivity analysis with the SMI diagnosis date based on the earliest record of the most severe SMI)


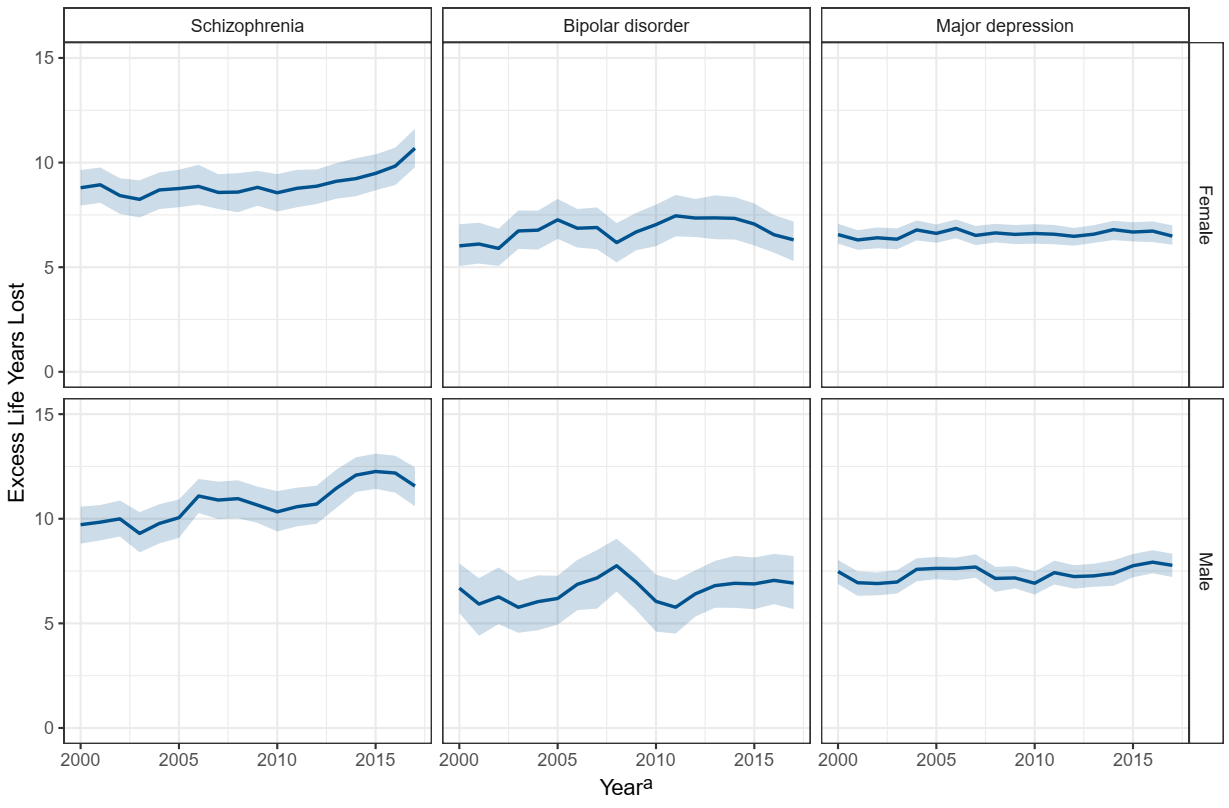


a. at the start of three-year period

# Supplementary Table 3: Life years lost for all deaths and natural and unnatural deaths among people in Scotland with each severe mental illness, stratified by sex (rolling three-year averages between 2000 and 2019) (sensitivity analysis with the SMI diagnosis date based on the earliest record of the most severe SMI)

|  |  |  | **SMI** | | |
| --- | --- | --- | --- | --- | --- |
| **Cause of death** | **Sex** | **Period** | **Schizophrenia** | **Bipolar disorder** | **Major depression** |
| All | Male | 2000-02 | 29.1 (28.2, 29.9) | 24.8 (23.6, 26.0) | 25.0 (24.4, 25.5) |
|  |  | 2001-03 | 29.0 (28.2, 29.9) | 23.9 (22.4, 25.1) | 24.3 (23.7, 24.8) |
|  |  | 2002-04 | 29.0 (28.1, 29.8) | 24.0 (22.7, 25.4) | 24.0 (23.5, 24.6) |
|  |  | 2003-05 | 27.9 (27.0, 28.9) | 23.2 (22.0, 24.5) | 23.8 (23.3, 24.4) |
|  |  | 2004-06 | 28.0 (27.1, 29.0) | 23.1 (21.8, 24.4) | 24.1 (23.5, 24.6) |
|  |  | 2005-07 | 28.1 (27.2, 29.0) | 23.1 (21.8, 24.2) | 24.0 (23.4, 24.5) |
|  |  | 2006-08 | 29.0 (28.2, 29.8) | 23.6 (22.3, 24.7) | 23.8 (23.2, 24.3) |
|  |  | 2007-09 | 28.5 (27.6, 29.4) | 23.6 (22.1, 24.9) | 23.6 (23.1, 24.2) |
|  |  | 2008-10 | 28.2 (27.3, 29.1) | 23.9 (22.6, 25.2) | 22.7 (22.1, 23.3) |
|  |  | 2009-11 | 27.6 (26.7, 28.5) | 22.8 (21.4, 24.1) | 22.5 (22.0, 23.0) |
|  |  | 2010-12 | 27.0 (26.1, 28.0) | 21.7 (20.2, 22.9) | 22.0 (21.5, 22.6) |
|  |  | 2011-13 | 27.1 (26.1, 28.0) | 21.2 (20.0, 22.5) | 22.4 (21.8, 22.9) |
|  |  | 2012-14 | 27.0 (26.1, 27.9) | 21.7 (20.6, 22.8) | 22.0 (21.4, 22.6) |
|  |  | 2013-15 | 27.8 (26.8, 28.7) | 22.1 (21.0, 23.3) | 22.1 (21.5, 22.6) |
|  |  | 2014-16 | 28.4 (27.6, 29.3) | 22.2 (21.0, 23.5) | 22.2 (21.6, 22.8) |
|  |  | 2015-17 | 28.6 (27.8, 29.5) | 22.2 (20.9, 23.4) | 22.5 (22.0, 23.1) |
|  |  | 2016-18 | 28.5 (27.5, 29.3) | 22.2 (21.1, 23.5) | 22.6 (22.1, 23.2) |
|  |  | 2017-19 | 27.8 (26.8, 28.7) | 22.0 (20.8, 23.3) | 22.4 (21.8, 22.9) |
|  | Female | 2000-02 | 22.7 (21.8, 23.5) | 20.0 (19.0, 21.0) | 19.8 (19.4, 20.3) |
|  |  | 2001-03 | 22.8 (21.9, 23.6) | 20.0 (19.0, 21.0) | 19.5 (19.0, 19.9) |
|  |  | 2002-04 | 22.1 (21.2, 22.9) | 19.6 (18.8, 20.6) | 19.4 (18.9, 19.9) |
|  |  | 2003-05 | 21.7 (20.9, 22.7) | 20.3 (19.4, 21.3) | 19.2 (18.7, 19.7) |
|  |  | 2004-06 | 22.0 (21.1, 22.8) | 20.1 (19.2, 21.0) | 19.4 (18.9, 19.9) |
|  |  | 2005-07 | 21.9 (21.0, 22.8) | 20.5 (19.6, 21.5) | 19.2 (18.7, 19.6) |
|  |  | 2006-08 | 21.9 (21.0, 22.9) | 20.0 (19.0, 20.9) | 19.3 (18.8, 19.7) |
|  |  | 2007-09 | 21.4 (20.7, 22.3) | 19.8 (18.8, 20.8) | 18.8 (18.3, 19.2) |
|  |  | 2008-10 | 21.3 (20.3, 22.2) | 18.9 (18.0, 19.8) | 18.7 (18.3, 19.2) |
|  |  | 2009-11 | 21.3 (20.4, 22.0) | 19.2 (18.3, 20.1) | 18.4 (18.0, 18.9) |
|  |  | 2010-12 | 20.9 (20.0, 21.8) | 19.5 (18.4, 20.4) | 18.4 (17.9, 18.9) |
|  |  | 2011-13 | 21.1 (20.1, 21.9) | 19.8 (18.8, 20.8) | 18.3 (17.8, 18.7) |
|  |  | 2012-14 | 21.0 (20.2, 21.8) | 19.6 (18.7, 20.5) | 18.1 (17.6, 18.5) |
|  |  | 2013-15 | 21.2 (20.4, 22.1) | 19.5 (18.5, 20.6) | 18.1 (17.7, 18.6) |
|  |  | 2014-16 | 21.3 (20.5, 22.3) | 19.4 (18.4, 20.5) | 18.3 (17.8, 18.7) |
|  |  | 2015-17 | 21.6 (20.8, 22.5) | 19.2 (18.2, 20.2) | 18.2 (17.8, 18.7) |
|  |  | 2016-18 | 21.9 (21.0, 22.8) | 18.7 (17.8, 19.6) | 18.2 (17.7, 18.7) |
|  |  | 2017-19 | 22.7 (21.8, 23.7) | 18.4 (17.4, 19.3) | 18.0 (17.5, 18.5) |
| Natural | Male | 2000-02 | 24.7 (23.7, 25.6) | 21.1 (19.6, 22.4) | 20.3 (19.7, 20.9) |
|  |  | 2001-03 | 25.0 (24.1, 25.9) | 20.2 (18.8, 21.5) | 19.8 (19.2, 20.5) |
|  |  | 2002-04 | 24.8 (23.8, 25.7) | 19.9 (18.6, 21.3) | 19.8 (19.3, 20.4) |
|  |  | 2003-05 | 23.7 (22.7, 24.7) | 19.5 (18.1, 20.9) | 19.8 (19.3, 20.4) |
|  |  | 2004-06 | 23.9 (23.0, 24.8) | 19.6 (18.2, 20.9) | 20.1 (19.4, 20.7) |
|  |  | 2005-07 | 23.9 (22.9, 24.8) | 19.8 (18.5, 20.9) | 20.0 (19.5, 20.6) |
|  |  | 2006-08 | 24.3 (23.3, 25.2) | 19.8 (18.5, 21.2) | 20.1 (19.5, 20.7) |
|  |  | 2007-09 | 24.0 (23.1, 24.9) | 20.1 (18.8, 21.3) | 19.8 (19.2, 20.4) |
|  |  | 2008-10 | 23.8 (22.7, 24.7) | 20.3 (19.0, 21.8) | 19.1 (18.5, 19.6) |
|  |  | 2009-11 | 22.7 (21.8, 23.7) | 19.4 (18.0, 20.7) | 18.0 (17.5, 18.6) |
|  |  | 2010-12 | 21.3 (20.3, 22.3) | 18.4 (17.0, 19.7) | 17.4 (16.8, 17.9) |
|  |  | 2011-13 | 20.7 (19.7, 21.7) | 18.0 (16.7, 19.3) | 17.4 (16.9, 18.0) |
|  |  | 2012-14 | 21.1 (20.1, 22.0) | 18.6 (17.5, 19.7) | 17.3 (16.7, 17.8) |
|  |  | 2013-15 | 21.8 (21.0, 22.7) | 18.7 (17.6, 19.9) | 17.3 (16.7, 17.8) |
|  |  | 2014-16 | 22.2 (21.4, 23.0) | 18.3 (17.1, 19.5) | 16.9 (16.4, 17.5) |
|  |  | 2015-17 | 22.3 (21.4, 23.1) | 17.8 (16.6, 19.1) | 17.2 (16.7, 17.7) |
|  |  | 2016-18 | 21.2 (20.4, 22.1) | 17.4 (16.2, 18.6) | 16.8 (16.3, 17.4) |
|  |  | 2017-19 | 21.8 (20.9, 22.7) | 18.0 (16.7, 19.4) | 17.6 (17.0, 18.1) |
|  | Female | 2000-02 | 20.4 (19.4, 21.3) | 17.7 (16.7, 18.6) | 17.4 (17.0, 17.9) |
|  |  | 2001-03 | 20.1 (19.1, 21.0) | 17.2 (16.3, 18.2) | 16.9 (16.4, 17.4) |
|  |  | 2002-04 | 19.5 (18.6, 20.4) | 17.1 (16.2, 18.0) | 16.9 (16.4, 17.4) |
|  |  | 2003-05 | 19.5 (18.6, 20.4) | 17.4 (16.4, 18.3) | 16.6 (16.1, 17.1) |
|  |  | 2004-06 | 19.6 (18.6, 20.5) | 17.7 (16.8, 18.7) | 17.0 (16.5, 17.4) |
|  |  | 2005-07 | 19.2 (18.3, 20.2) | 18.1 (17.1, 19.0) | 16.8 (16.4, 17.2) |
|  |  | 2006-08 | 19.5 (18.6, 20.5) | 17.6 (16.7, 18.5) | 17.1 (16.7, 17.6) |
|  |  | 2007-09 | 19.2 (18.3, 20.1) | 16.7 (15.6, 17.7) | 16.7 (16.3, 17.2) |
|  |  | 2008-10 | 19.4 (18.4, 20.3) | 16.4 (15.4, 17.3) | 16.6 (16.2, 17.1) |
|  |  | 2009-11 | 18.7 (17.7, 19.6) | 16.7 (15.8, 17.7) | 16.0 (15.5, 16.4) |
|  |  | 2010-12 | 18.3 (17.5, 19.3) | 16.9 (15.9, 17.8) | 15.8 (15.3, 16.2) |
|  |  | 2011-13 | 18.2 (17.3, 19.1) | 16.8 (15.9, 17.7) | 15.8 (15.4, 16.2) |
|  |  | 2012-14 | 18.6 (17.7, 19.5) | 16.8 (15.8, 17.7) | 15.8 (15.4, 16.2) |
|  |  | 2013-15 | 18.7 (17.9, 19.6) | 16.8 (15.8, 17.7) | 15.5 (15.1, 15.9) |
|  |  | 2014-16 | 18.6 (17.8, 19.5) | 16.5 (15.6, 17.6) | 15.3 (14.9, 15.7) |
|  |  | 2015-17 | 18.6 (17.8, 19.5) | 15.9 (15.0, 16.8) | 15.1 (14.7, 15.6) |
|  |  | 2016-18 | 18.7 (17.8, 19.6) | 15.8 (15.0, 16.7) | 15.1 (14.7, 15.6) |
|  |  | 2017-19 | 20.0 (19.1, 21.0) | 15.7 (14.9, 16.5) | 15.4 (15.0, 15.9) |
| Unnatural | Male | 2000-02 | 4.4 (3.7, 5.2) | 3.7 (2.6, 4.9) | 4.7 (4.0, 5.2) |
|  |  | 2001-03 | 4.1 (3.4, 4.8) | 3.7 (2.5, 4.9) | 4.5 (3.8, 5.1) |
|  |  | 2002-04 | 4.2 (3.4, 5.0) | 4.1 (2.9, 5.3) | 4.2 (3.7, 4.7) |
|  |  | 2003-05 | 4.3 (3.5, 5.1) | 3.7 (2.7, 5.0) | 4.0 (3.5, 4.6) |
|  |  | 2004-06 | 4.2 (3.4, 5.0) | 3.6 (2.5, 4.8) | 4.0 (3.5, 4.6) |
|  |  | 2005-07 | 4.2 (3.5, 4.9) | 3.3 (2.3, 4.4) | 3.9 (3.4, 4.4) |
|  |  | 2006-08 | 4.7 (3.9, 5.4) | 3.8 (2.8, 4.9) | 3.7 (3.1, 4.2) |
|  |  | 2007-09 | 4.5 (3.8, 5.2) | 3.5 (2.4, 4.7) | 3.8 (3.3, 4.3) |
|  |  | 2008-10 | 4.4 (3.7, 5.1) | 3.5 (2.5, 4.7) | 3.6 (3.1, 4.1) |
|  |  | 2009-11 | 4.9 (4.1, 5.7) | 3.3 (2.3, 4.5) | 4.4 (4.0, 5.0) |
|  |  | 2010-12 | 5.7 (4.9, 6.6) | 3.2 (2.2, 4.2) | 4.6 (4.1, 5.2) |
|  |  | 2011-13 | 6.3 (5.5, 7.2) | 3.2 (2.2, 4.2) | 4.9 (4.4, 5.5) |
|  |  | 2012-14 | 5.9 (5.1, 6.8) | 3.1 (2.2, 4.0) | 4.8 (4.2, 5.3) |
|  |  | 2013-15 | 6.0 (5.0, 6.9) | 3.4 (2.6, 4.5) | 4.8 (4.2, 5.4) |
|  |  | 2014-16 | 6.2 (5.2, 7.1) | 3.9 (2.8, 4.9) | 5.2 (4.6, 5.8) |
|  |  | 2015-17 | 6.3 (5.4, 7.4) | 4.3 (3.1, 5.5) | 5.3 (4.8, 6.0) |
|  |  | 2016-18 | 7.3 (6.3, 8.2) | 4.8 (3.8, 6.0) | 5.8 (5.2, 6.4) |
|  |  | 2017-19 | 6.0 (5.0, 6.9) | 4.0 (2.9, 5.3) | 4.8 (4.3, 5.4) |
|  | Female | 2000-02 | 2.3 (1.7, 2.9) | 2.3 (1.5, 3.1) | 2.3 (2.0, 2.7) |
|  |  | 2001-03 | 2.7 (2.0, 3.4) | 2.7 (1.9, 3.6) | 2.5 (2.2, 2.9) |
|  |  | 2002-04 | 2.6 (1.9, 3.3) | 2.5 (1.8, 3.4) | 2.5 (2.2, 2.9) |
|  |  | 2003-05 | 2.3 (1.7, 2.9) | 2.9 (2.1, 3.7) | 2.6 (2.2, 2.9) |
|  |  | 2004-06 | 2.4 (1.8, 3.1) | 2.4 (1.7, 3.1) | 2.5 (2.1, 2.8) |
|  |  | 2005-07 | 2.7 (2.0, 3.5) | 2.4 (1.7, 3.1) | 2.4 (2.1, 2.7) |
|  |  | 2006-08 | 2.4 (1.7, 3.1) | 2.4 (1.7, 3.1) | 2.1 (1.8, 2.5) |
|  |  | 2007-09 | 2.2 (1.7, 2.8) | 3.1 (2.3, 3.9) | 2.1 (1.8, 2.4) |
|  |  | 2008-10 | 1.9 (1.3, 2.5) | 2.5 (1.7, 3.3) | 2.1 (1.8, 2.4) |
|  |  | 2009-11 | 2.6 (1.9, 3.2) | 2.5 (1.8, 3.2) | 2.4 (2.1, 2.8) |
|  |  | 2010-12 | 2.6 (1.9, 3.3) | 2.6 (1.8, 3.4) | 2.6 (2.2, 2.9) |
|  |  | 2011-13 | 2.8 (2.2, 3.6) | 3.0 (2.2, 3.8) | 2.5 (2.1, 2.8) |
|  |  | 2012-14 | 2.4 (1.8, 3.1) | 2.8 (2.1, 3.6) | 2.3 (2.0, 2.6) |
|  |  | 2013-15 | 2.5 (1.8, 3.2) | 2.7 (2.0, 3.5) | 2.6 (2.3, 3.0) |
|  |  | 2014-16 | 2.7 (2.0, 3.5) | 3.0 (2.2, 3.8) | 3.0 (2.6, 3.4) |
|  |  | 2015-17 | 3.0 (2.2, 3.8) | 3.3 (2.5, 4.2) | 3.1 (2.7, 3.5) |
|  |  | 2016-18 | 3.2 (2.4, 4.0) | 2.8 (2.0, 3.7) | 3.1 (2.7, 3.5) |
|  |  | 2017-19 | 2.7 (2.0, 3.5) | 2.7 (2.0, 3.5) | 2.5 (2.1, 2.9) |

# Supplementary Table 4: Excess life years lost in people with a severe mental illness in comparison to the Scottish population, stratified by sex (rolling three-year averages between 2000 and 2019) (sensitivity analysis with the SMI diagnosis date based on the earliest record of the most severe SMI)

|  |  | **SMI** | | |
| --- | --- | --- | --- | --- |
| **Sex** | **Period** | **Schizophrenia** | **Bipolar disorder** | **Depression** |
| Male | 2000-02 | 9.7 (8.8, 10.6) | 6.7 (5.5, 7.9) | 7.5 (6.9, 8.0) |
|  | 2001-03 | 9.8 (9.0, 10.7) | 5.9 (4.4, 7.2) | 6.9 (6.3, 7.5) |
|  | 2002-04 | 10.0 (9.2, 10.9) | 6.3 (5.0, 7.7) | 6.9 (6.3, 7.4) |
|  | 2003-05 | 9.3 (8.4, 10.3) | 5.8 (4.5, 7.0) | 7.0 (6.4, 7.5) |
|  | 2004-06 | 9.8 (8.8, 10.7) | 6.0 (4.7, 7.3) | 7.6 (7.0, 8.1) |
|  | 2005-07 | 10.1 (9.1, 10.9) | 6.2 (4.9, 7.3) | 7.6 (7.1, 8.2) |
|  | 2006-08 | 11.1 (10.3, 11.9) | 6.9 (5.6, 8.0) | 7.6 (7.1, 8.1) |
|  | 2007-09 | 10.9 (10.0, 11.8) | 7.2 (5.7, 8.5) | 7.7 (7.2, 8.3) |
|  | 2008-10 | 11.0 (10.0, 11.8) | 7.8 (6.5, 9.0) | 7.1 (6.5, 7.7) |
|  | 2009-11 | 10.7 (9.8, 11.5) | 7.0 (5.6, 8.2) | 7.2 (6.7, 7.7) |
|  | 2010-12 | 10.3 (9.4, 11.3) | 6.1 (4.6, 7.3) | 6.9 (6.4, 7.5) |
|  | 2011-13 | 10.6 (9.6, 11.5) | 5.8 (4.5, 7.1) | 7.4 (6.9, 8.0) |
|  | 2012-14 | 10.7 (9.8, 11.6) | 6.4 (5.3, 7.5) | 7.2 (6.7, 7.8) |
|  | 2013-15 | 11.5 (10.5, 12.3) | 6.8 (5.7, 8.0) | 7.3 (6.7, 7.8) |
|  | 2014-16 | 12.1 (11.3, 12.9) | 6.9 (5.7, 8.2) | 7.4 (6.8, 8.0) |
|  | 2015-17 | 12.3 (11.4, 13.1) | 6.9 (5.7, 8.1) | 7.8 (7.2, 8.3) |
|  | 2016-18 | 12.2 (11.2, 13.0) | 7.1 (5.9, 8.3) | 7.9 (7.4, 8.5) |
|  | 2017-19 | 11.6 (10.6, 12.5) | 6.9 (5.7, 8.2) | 7.8 (7.2, 8.3) |
| Female | 2000-02 | 8.8 (7.9, 9.6) | 6.0 (5.1, 7.0) | 6.6 (6.1, 7.1) |
|  | 2001-03 | 8.9 (8.1, 9.8) | 6.1 (5.2, 7.1) | 6.3 (5.8, 6.8) |
|  | 2002-04 | 8.4 (7.5, 9.3) | 5.9 (5.1, 6.8) | 6.4 (5.9, 6.9) |
|  | 2003-05 | 8.2 (7.4, 9.2) | 6.7 (5.9, 7.7) | 6.3 (5.9, 6.9) |
|  | 2004-06 | 8.7 (7.8, 9.5) | 6.8 (5.8, 7.7) | 6.8 (6.3, 7.2) |
|  | 2005-07 | 8.8 (7.9, 9.7) | 7.3 (6.4, 8.3) | 6.6 (6.2, 7.0) |
|  | 2006-08 | 8.9 (8.0, 9.9) | 6.9 (5.9, 7.8) | 6.9 (6.4, 7.3) |
|  | 2007-09 | 8.6 (7.8, 9.4) | 6.9 (5.9, 7.9) | 6.5 (6.1, 7.0) |
|  | 2008-10 | 8.6 (7.6, 9.5) | 6.2 (5.2, 7.1) | 6.6 (6.2, 7.1) |
|  | 2009-11 | 8.8 (7.9, 9.6) | 6.7 (5.8, 7.6) | 6.6 (6.1, 7.0) |
|  | 2010-12 | 8.6 (7.7, 9.5) | 7.0 (6.0, 8.0) | 6.6 (6.1, 7.1) |
|  | 2011-13 | 8.8 (7.9, 9.7) | 7.5 (6.5, 8.5) | 6.6 (6.1, 7.0) |
|  | 2012-14 | 8.9 (8.0, 9.7) | 7.3 (6.4, 8.3) | 6.5 (6.0, 6.9) |
|  | 2013-15 | 9.1 (8.3, 10.0) | 7.4 (6.3, 8.4) | 6.6 (6.2, 7.0) |
|  | 2014-16 | 9.2 (8.4, 10.2) | 7.3 (6.3, 8.4) | 6.8 (6.3, 7.2) |
|  | 2015-17 | 9.5 (8.7, 10.4) | 7.1 (6.0, 8.0) | 6.7 (6.2, 7.1) |
|  | 2016-18 | 9.8 (8.9, 10.7) | 6.6 (5.7, 7.5) | 6.7 (6.2, 7.2) |
|  | 2017-19 | 10.7 (9.8, 11.6) | 6.3 (5.3, 7.2) | 6.5 (6.1, 7.0) |
